# Effects of Daily Consumption of Soy Products on Basic/Instrumental Activities of Daily Living in Community-Dwelling Japanese Women Aged 75 Years and Older: A 4-Year Cohort Study

**DOI:** 10.1089/whr.2022.0076

**Published:** 2023-05-15

**Authors:** Narumi Kojima, Miji Kim, Kyoko Saito, Yuko Yoshida, Hirohiko Hirano, Shuichi Obuchi, Hiroyuki Shimada, Takao Suzuki, Hajime Iwasa, Hunkyung Kim

**Affiliations:** ^1^ Research Team for Promoting Independence and Mental Health, Tokyo Metropolitan Institute of Gerontology, Tokyo, Japan.; ^2^ Department of Biomedical Science and Technology, College of Medicine, East-West Medical Research Institute, Kyung Hee University, Seoul, Republic of Korea.; ^3^ Graduate School of Medicine, Yokohama City University, Yokohama, Japan.; ^4^ Research Team for Human Care, Tokyo Metropolitan Institute of Gerontology, Tokyo, Japan.; ^5^ National Center for Geriatrics and Gerontology, Research Institute, Obu-shi, Japan.; ^6^ Department of Public Health, Fukushima Medical University, Fukushima City, Japan.

**Keywords:** basic activities of daily living, instrumental activities of daily living, isoflavones, soy products,

## Abstract

**Introduction:**

Since soy isoflavones compensate for age-related estrogen reduction, adequate intake of soy products may prevent the decline in activities of daily living (ADL) due to estrogen reduction in women. However, it is unclear whether regular soy product intake prevents ADL decline. This study examined the effects of soy product consumption on basic/instrumental ADL (BADL/IADL) in Japanese women 75 years or older for 4 years.

**Materials and Methods:**

The subject population consisted of 1289 women aged 75 years or older living in Tokyo who underwent private health examinations in 2008. For 1114 (or 1042) participants without baseline BADL (or IADL) disability, we examined the association between baseline soy product consumption frequency and the BADL (or IADL) disabilities 4 years later using logistic regression analyses. The models were adjusted for baseline age, or further for dietary variety for food groups other than soy products, exercise and sport participation, smoking, pre-existing disease number, and body mass index.

**Results:**

Regardless of adjustment for potential confounding factors, less frequent soy product consumption was associated with higher BADL or IADL disability incidence. In the fully adjusted models, the trend toward a higher incidence of disabilities with less frequent soy product consumption was statistically significant for both BADL (*p* = 0.001) and IADL (*p* = 0.007).

**Conclusions:**

Those who consumed soy products more frequently at baseline were less likely to develop BADL and IADL disabilities after 4 years than those who did not. The results show that daily soy product consumption may prevent functional ADL decline in older Japanese women.

## Introduction

In Japan, the decline of activities of daily living (ADL) has been a major problem in older people, especially among women >75 years of age. Annual data for FY 2015 by the Japan Sports Agency compares ADLs that consist of 12 items, by age and gender.^1^ According to their data, women, especially in the 75–79 age group, tend to experience a noticeable decline in ADLs involving gross movements that require muscle strength. Women aged 75–79 years tend to have weaker physical function in tasks, such as walking continuously for prolonged time, climbing stairs without holding on to handrails, remaining standing on buses and trains, and wearing a pant or skirt while standing, than women aged 70–74 years or men aged 75–79 years.^1^

Prevention of functional decline is thus an urgent issue for the older people, especially for women >75 years old in Japan. This is not limited to Japan, as evident by the United States National Health Interview Survey (2011), reporting that ∼7% individuals aged 75–84 years needed help with ADLs, whereas only 3.4% of those aged 65–74 years required the same.^2^

This decline in life function in older women may be partly due to the increased incidence of various diseases related to decreased estrogen secretion. The secretion of estrogen, a type of female hormone, increases around puberty, and then declines sharply after ∼10-year menopausal period around age 50 years.^3^ It has been reported that the lowered estrogen levels not only cause menopausal symptoms such as hot flashes, irritability, and depression, but also increase the risk of various diseases such as osteoporosis, stroke, atherosclerosis, ischemic heart disease, and Alzheimer's disease during old age.^4^

Soybeans, which are processed into fermented or nonfermented foods for daily consumption, occupy a very important place in Japanese food culture. Several epidemiological reports support the theoretical possibility that soy isoflavone intake may compensate for this decreased estrogen secretion and contribute to the prevention of various female aging diseases.^5,6^ Isoflavones such as daidzein, genistin, and glycitin are a type of flavonoid produced by plants for self-defense purposes and are found mainly in the soybean germ. Isoflavone is also called “phytoestrogen” because of its molecular structure similarity to estrogen, its ability to bind to estrogen receptors, and its female hormone-like effects.

Isoflavones also have antiestrogenic effects, that is, they exert a regulatory function that normalizes estrogen function.^5,6^ There have been several reports of isoflavone intake contributing to the disease prevention. As far as postmenopausal women are concerned, epidemiological evidence suggests that dietary soy intake decreases the risk of breast cancer.^5^ Epidemiological studies and clinical trials also suggest that soy isoflavones have beneficial effects on bone mineral density, bone metabolism, and bone mechanical strength in postmenopausal women, contributing to the prevention and treatment of postmenopausal osteoporosis.^6^

In addition to disease-preventive effects, a cross-sectional study in Korea reported that frequent consumption of soy beans and soy products was inversely associated with instrumental ADL (IADL) impairment in older women.^7^ To our knowledge, however, there are no studies that have longitudinally investigated the association between the frequency of soy product consumption and the occurrence of IADL and basic ADL (BADL) disabilities.

In this study, we examined whether the consumption of soy products was associated with the prevention of decline in ADL over several years in Japanese women aged 75 years and older. The endpoint was the incidence of BADL and IADL disabilities after 4 years.

## Materials and Methods

### Study design

This is a 4-year cohort study conducted as a follow-up of community-dwelling women without baseline BADL or IADL disabilities. Baseline and follow-up measurements were conducted in October–November 2008 and October 2012, respectively. The main independent variable was the consumption frequency of soy products at baseline, whereas the dependent variable was BADL or IADL disabilities at follow-up.

### Participants

In the summer of 2008, we mailed flyers to randomly selected homes in the southeastern part of Itabashi Ward, Tokyo, to invite women whose date of birth was between October 1, 1923, and November 30, 1933, to participate in our private health checkup event. As a result, 1289 women participated in the baseline survey held between October 15 and November 3, 2008. Of the 1289 participants, 10 had baseline BADL disability, and 1 lacked information on BADL, resulting in 1278 participants without baseline BADL disability.

Of the 1289 participants, 64 had baseline IADL disability, and 1 lacked information on IADL, resulting in 1224 participants without baseline IADL disability. Researchers conducted follow-up surveys in October 2012, either on-site or, for those who were unable to participate on-site, *via* mail. Of the 1289 baseline participants, 575 participated in the on-site survey and 666 participated in the mail survey, resulting in 1241 (96%) participants to be analyzed. The ethics committee of the author's affiliated institute approved the study protocol. All participants provided written informed consent regarding academic usage of their data.

### Setting

Researchers conducted the baseline data collection through on-site interviews at the author's affiliated research institute in Itabashi Ward, Tokyo. The follow-up data collection was conducted through on-site interviews at our institute or through mail surveys. The interval (mean and standard deviation) between the baseline and the follow-up data collections was 1462.7 (5.4) days.

### Measurements

At the baseline data collection, researchers determined the health status and lifestyle of the participants, including the frequency of soy product consumption and BADL/IADL status, through structured interviews conducted by trained research associates with no prior knowledge about each participant's profile. During the follow-up data collection, the researchers determined BADL/IADL status through interviews for the on-site participants and mail surveys for the others.

### Functional disability questions

Functional ability was assessed based on the capacity to perform BADL and IADL. The “modified Katz Index” was used to assess BADL, which is a version of the Katz Index with modifications to take Japanese culture into account. The original Katz Index rates the level of independence in each of the six categories: bathing, dressing, toileting, transferring, continence, and feeding, on an integer scale of 0 (least independent) to 2 (most independent), with the higher total score indicating greater independence.^8^ We adopted five items from the original Katz Index, except for the one on continence, and used them to measure BADL, with the details of the wording modified to fit the Japanese culture. The BADL items we used were as follows:

We would like to ask you about your ADL. Please answer the number that applies to each item. (Please choose only one option.)

A. “Are you able to walk independently on your own? You may use a cane if necessary.”
  1. “I can walk.” or “I can walk if slowly.”
  2. “I can walk if I hold on to a handrail, etc.” or “I can walk if I am assisted.”
  3. “I cannot walk.”
B. “Can you eat meals by yourself?”
  1. “I can eat by myself. I do not need any special care.”
  2. “I cannot eat unless someone makes it easier for me, such as by breaking up fish or cutting meat into small pieces.”
  3. “I cannot eat by myself.”
C. “Can you bathe by yourself?”
  1. “Yes, I can. No special care is needed.”
  2. “I need partial assistance to get in and out of the bathtub and to wash myself.”
  3. “I cannot bathe without full assistance.” or “I can only be wiped clean because I cannot bathe.”
D. “Can you change clothes by yourself?”
  1. “Yes, it may take some time, but I can change by myself.”
  2. “I need help with buttoning and doing up a sash.”
  3. “I cannot change my clothes without full assistance.”
E. “Are you able to use the toilet by yourself?”
  1. “Yes, I can. I do not need any special consideration.”
  2. “I can use the toilet with assistance.” or “I use a portable toilet.”
  3. “I always use diapers or defecate on my bed.”

Two points were given for answer 1, one point for answer 2, and zero for answer 3, and the total score (0–10) was used to evaluate the BADLs. BADL disability was operationally defined as the inability to implement at least one of the mentioned five activities without assistance. That is, only if the score was 10, the patient was judged to have no BADL disability.

IADL performance was assessed using a five-item list from the TMIG (Tokyo Metropolitan Institute of Gerontology) Index of Competence for instrumental self-maintenance, which was developed for Japanese people and has been widely used in Japanese communities.^9^ The list assessed the following five activities: (1) using public transportation, (2) shopping for daily necessities, (3) preparing meals, (4) paying bills, and (5) handling a bank account. The response to each item was either “yes” (able to accomplish, 1 point) or “no” (unable to accomplish, 0 points). The IADL score was calculated as the total number of points (0–5). The IADL questions we used were as follows:

TMIG instrumental activities of daily living

**Table:** 

Question	Answer
i. Can you use public transportation (bus or train) by yourself?	Yes/No
ii. Are you able to shop for daily necessities?	Yes/No
iii. Are you able to prepare meals by yourself?	Yes/No
iv. Are you able to pay bills?	Yes/No
v. Can you handle your own banking?	Yes/No

IADL disability was defined as the inability to implement at least one of the mentioned five activities (score: 0–4).

### Soy product consumption frequency and the food variety questions

Baseline soy product intake frequency was measured using a food frequency questionnaire, which was originally developed by Kumagai et al^10^ for the purpose of measuring dietary variety in the Japanese population. The frequency of consumption of each 10 food groups (seafood, meat, eggs, milk, soy products, colored vegetables, seaweed, potatoes, fruits, and fats/oils) was measured using this questionnaire. Subjects were asked to choose how often during the past 1 week or so they consumed each food group from the following options: (1) almost every day, (2) once every 2 days, (3) once or twice a week, or (4) almost never.

The food group names were presented with a supplementary explanation in parentheses as follows: “A. Seafood (raw or processed: all fish and shellfish); B. Meat (raw or processed: all meat); C. Eggs (chicken eggs and quail eggs. Fish eggs should be classified as “A. Seafood”); D. Milk (excluding coffee/fruit-flavored milk); E. Soy products (foods using soybeans, such as tofu and natto); F. Colored vegetables (dark vegetables such as carrots, spinach, pumpkins, and tomatoes); G. Seaweed (regardless of raw or dried); H. Potatoes; I. Fruits (regardless of fresh or canned. Tomatoes should be classified as “F. Colored vegetables”); and J. Fats/oils (count dishes that use oil, such as stir-fry, butter- or margarine-coated bread).”

### Other questions or items related to potential confounders

The following items were measured in the baseline measurement as potential confounders in functional changes over time.

-Age
-Exercise and sports habits (yes/no)
-Current smoking habit (yes/no)
-Disease history (yes/no; hypertension, stroke, heart disease, diabetes, hyperlipidemia, osteoporosis, anemia, bronchial asthma, chronic obstructive pulmonary disease, hip osteoarthritis, knee osteoarthritis, and fracture since age 60 years)
-Height and weight were measured to calculate body mass index (BMI).

### Statistical analysis

Explanatory or control variables at baseline were defined as follows.

-Frequency of soy product consumption was coded as “almost every day (reference),” “once every 2 days,” “once or twice a week,” and “almost never.”
-Age was calculated based on the birthday and the date of the baseline data collection.
-A food variety index for the 9 (original 10 minus “soy products”) food groups (*i.e.*, the number of the food groups consumed almost every day, 0–9) was calculated and was defined as “partial dietary variety score (P-DVS).” The reason for not including soy products as the constructs of the food variety index was to prevent theoretical multicollinearity between soy product consumption frequency and the food variety index.
-Exercise and sports habits were coded as “no (reference)” or “yes.”
-Current smoking habit was coded as “no (reference)” or “yes.”
-The number of pre-existing diseases was counted.

The incidence of BADL or IADL disability at follow-up, depending on the baseline consumption frequency of soy products, was examined through logistic regression analyses. In Model 1, baseline age was incorporated into the regression model as a control variable using the forced entry method. In Model 2, baseline variables that we hypothesized could potentially affect both soy product consumption and BADL/IADL: regular exercise and sports, the number of pre-existing diseases, smoking habit, the P-DVS, and binarized BMI (overweight; BMI ≥25.0 kg/m^2^ or normal; BMI <25.0 kg/m^2^)^11^ were also incorporated into the regression model.

To examine trends in the incidence of BADL/IADL disability as a function of soy product consumption frequency, trend tests (Mantel extension tests) were performed with the frequency of consumption categories considered as a quantitative variable of 1, 2, 3, or 4. All statistical analyses were conducted using SPSS Statistics 26.0 (SPSS, Inc., Chicago, IL, USA).

## Results

### Basic characteristics of the participants

Of the 1289 baseline participants, 1278 had no and 10 did have baseline BADL disability, whereas the BADL data were missing for 1 participant. Of the 1278 participants without baseline BADL disability, the BADL data at follow-up were lacking for 164 participants, resulting in 1114 complete pairs of baseline and follow-up data on BADL disability.

Of the 1289 baseline participants, 1224 had no, and 64 had baseline IADL disability, whereas the IADL data were missing for 1 participant. Of the 1224 participants without baseline IADL disability, the IADL data at follow-up was lacking for 182 participants, resulting in 1042 complete pairs of baseline and follow-up data on IADL disability.

The baseline characteristics (age, height, weight, BMI, soy product intake, regular exercise and sports, the number of pre-existing diseases, smoking habits, and the P-DVS) of the total 1289 participants, the 1114 participants without baseline BADL disability, and with BADL data at follow-up, and the 1042 participants without baseline IADL disability and with IADL data at follow-up, are given in Table 1.

**Table 1.** Baseline Characteristics of the Participants

	Total participants			Participants without baseline BADL disability			Participants without baseline IADL disability		
	***N***	Mean	SD	***N***	Mean	SD	***N***	Mean	SD
Age, years	1289	78.5	2.7	1114	78.4	2.7	1042	78.3	2.6
Height, cm	1289	147.9	5.5	1114	148.0	5.4	1042	148.2	5.3
Weight, kg	1289	49.8	7.9	1114	49.9	7.9	1042	50.0	7.9
BMI (kg/m^2^)	1289	22.7	3.3	1114	22.8	3.3	1042	22.8	3.3
No. of pre-existing diseases	1289	2.2	1.4	1114	2.2	1.4	1042	2.2	1.4
P-DVS	1288	4.9	1.8	1114	4.9	1.8	1042	4.9	1.8

	***N***	**Yes**	**%**	***N***	**Yes**	**%**	***N***	**Yes**	%
Regular exercise and sports	1288	410	31.8	1114	369	33.1	1042	363	34.8
Present or past smoking	1288	127	9.9	1114	105	9.4	1042	100	9.6
BMI (kg/m^2^) ≥25	1289	300	23.3	1042	242	23.2	1042	242	23.2
Soy products	1288			1114			1042		
Almost every day		864	67.1		747	67.1		706	67.8
Once every 2 days		239	18.6		199	17.9		186	17.9
Once or twice a week		157	12.2		144	12.9		130	12.5
Almost never		28	2.2		24	2.2		20	1.9

BADL, basic activities of daily living; BMI, body mass index; IADL, instrumental activities of daily living; P-DVS, partial dietary variety score; SD, standard deviation

### The incidence of BADL or IADL disability at follow-up

Of the 1114 participants without BADL disability at baseline, 87 (7.8%) had BADL disability at follow-up, whereas 1027 (92.2%) did not and the incidence of BADL disability calculated using 2-point snapshots was 7.8%. Similarly, of the 1042 participants without IADL disability at baseline, 134 (12.9%) had IADL disability at follow-up, whereas 908 (87.1%) did not and the incidence of IADL disability was 12.9%.

### Factors associated with the incidence of BADL/IADL disabilities

Based on Model 2 (fully adjusted model), the variables associated with the incidence of BADL disability were frequency of soy product intake (*p* for trend = 0.001), age (*p* < 0.001), and the number of pre-existing diseases (*p* = 0.008) (Tables 2 and 3). The incidence of BADL disability increased with less frequent intake of soy products, older age, and a greater number of pre-existing diseases. Similarly, variables associated with the incidence of IADL disability were soy product intake (*p* for trend = 0.007), age (*p* < 0.001), and regular exercise and sports participation (*p* = 0.002). The incidence of IADL disability increased with less frequent intake of soy products, with older age, and with no regular exercise and sports.

**Table 2.** Baseline Factors Affecting Basic Activities of Daily Living Disability Incidence as Per Linear Regression

	Model 1			Model 2		
	***p***	OR	95% CI	***p***	OR	95% CI
Soy products (/+1 level toward lesser consumption)	0.002	1.47	1.16–1.87	0.001	1.51	1.18–1.94
Soy products (“almost every day” for reference)						
Once every 2 days	0.064	1.70	0.97–2.98	0.042	1.82	1.02–3.25
Once or twice a week	0.006	2.27	1.27–4.04	0.003	2.54	1.38–4.67
Almost never	0.099	2.62	0.84–8.24	0.115	2.54	0.80–8.07
Age (/+1 year)	<0.001	1.21	1.11–1.31	<0.001	1.19	1.10–1.29
P-DVS (/+1 point)				0.109	1.11	0.98–1.27
Regular exercise and sports (yes)				0.077	0.60	0.34–1.06
Present or past smoking (yes)				0.762	1.12	0.53–2.37
No. of pre-existing diseases (/+1)				0.008	1.23	1.06–1.44
BMI (kg/m^2^) ≥25 (yes)				0.553	0.84	0.48–1.48

Multivariate logistic regression (forced entry) was used to calculate ORs and 95% CIs. In Model 1, frequency of intake of soy products (either as a continuous or as a categorical variable) and age were incorporated. In Model 2, in addition to frequency of intake of soy products and age, P-DVS, regular exercise and sports, present or past smoking, number of pre-existing diseases, and BMI were also incorporated. The *p*-values, ORs, and 95% CIs, except for the first row [“Soy products (/+1 level toward lesser consumption)”], are for when the frequency of soy product intake is treated as a categorical variable

CI, confidence interval; OR, odds ratio.

**Table 3.** Baseline Factors Affecting Instrumental Activities of Daily Living Disability Incidence as Per Linear Regression

	**Model 1**			**Model 2**		
	***p***	**OR**	**95% CI**	***p***	**OR**	**95% CI**
Soy products (/+1 level toward lesser consumption)	0.005	1.36	1.10–1.68	0.007	1.37	1.09–1.72
Soy products (“almost every day” for reference)						
Once every 2 days	0.063	1.56	0.98–2.50	0.040	1.67	1.02–2.71
Once or twice a week	0.003	2.13	1.29–3.53	0.003	2.24	1.31–3.81
Almost never	0.783	1.20	0.33–4.39	0.874	1.11	0.30–4.13
Age (/+1 year)	<0.001	1.25	1.17–1.34	<0.001	1.24	1.15–1.33
P-DVS (/+1 point)				0.153	1.09	0.97–1.22
Regular exercise and sports (yes)				0.002	0.47	0.29–0.75
Present or past smoking (yes)				0.417	1.29	0.70–2.40
No. of pre-existing diseases(/+1)				0.135	1.11	0.97–1.26
BMI (kg/m^2^) ≥25 (yes)				0.713	0.92	0.58–1.46

Multivariate logistic regression (forced entry) was used to calculate ORs and 95% CIs. In Model 1, frequency of intake of soy products (either as a continuous or as a categorical variable) and age were incorporated. In Model 2, in addition to frequency of intake of soy products and age, P-DVS, regular exercise and sports, present or past smoking, number of pre-existing diseases, and BMI were also incorporated. The *p*-values, ORs, and 95% CIs, except for the first row [“Soy products (/+1 level toward lesser consumption)”], are for when the frequency of soy product intake is treated as a categorical variable.

### Effects of baseline consumption frequency of soy products on the incidence of BADL disability

In both Model 1, which was adjusted for age alone, and Model 2, which was also adjusted for other potential confounders, a lower frequency of soy product consumption was associated with a higher incidence of BADL disability (Table 2). Based on Model 1, the odds ratios (95% confidence intervals [CIs], *p*-values) for the incidence of BADL disability for subjects who consumed soy products “once every 2 days,” “once or twice a week,” and “almost never” were 1.70 (0.97–2.98, *p* = 0.064), 2.27 (1.27–4.04, *p* = 0.006), and 2.62 (0.84–8.24, *p* = 0.099), respectively, when those who consumed soy products “almost every day” were set as a reference. The trend toward a reduced incidence of BADL disability with more frequent consumption of soy products was statistically significant (*p* = 0.002).

Based on Model 2, the odds ratios (95% CIs) for the incidence of BADL disability for subjects who consumed soy products “once every 2 days,” “once or twice a week,” and “almost never” were 1.82 (1.02–3.25, *p* = 0.042), 2.54 (1.38–4.67, *p* = 0.003), and 2.54 (0.80–8.07, *p* = 0.115), respectively, when those who consumed soy products “almost every day” was set as a reference. The trend toward a higher incidence of BADL disability with less frequent consumption of soy products was statistically significant (*p* = 0.001).

### Effects of baseline consumption frequency of soy products on the incidence of IADL disability

In both Model 1 and Model 2, lower frequency of soy product consumption was associated with a higher incidence of IADL disability (Table 3). Based on Model 1, the odds ratios (95% CIs) for the incidence of IADL disability for subjects who consumed soy products “once every 2 days,” “once or twice a week,” and “almost never” were 1.56 (0.98–2.50, *p* = 0.063), 2.13 (1.29–3.53, *p* = 0.003), and 1.20 (0.33–4.39, *p* = 0.783), respectively, when those who consumed soy products “almost every day” were used as reference. The trend toward a higher incidence of IADL disability with less frequent consumption of soy products was statistically significant (*p* = 0.005).

Based on Model 2, the odds ratios (95% CIs) for the incidence of IADL disability for subjects who consumed soy products “once every 2 days,” “once or twice a week,” and “almost never” were 1.67 (1.02–2.71, *p* = 0.040), 2.24 (1.31–3.81, *p* = 0.003), and 1.11 (0.30–4.13, *p* = 0.874), respectively, when those who consumed soy products “almost every day” were set as a reference. The trend toward a higher incidence of IADL disability with less frequent consumption of soy products was statistically significant (*p* = 0.007).

## Discussion

The results showed that among community-dwelling Japanese women aged 75 years or older without BADL/IADL disability, those who consumed soy products more often had a lower probability of developing BADL/IADL disability 4 years later. These results were consistent when adjusted for age, regular exercise, and sports participation, the number of pre-existing diseases, smoking habit, the P-DVS, and BMI. This suggests that daily consumption of soy products has independent and beneficial effects on the maintenance of ADL in older Japanese women.

### Potential mechanisms linking soy products consumption and functional ability

BADL and IADL consist of both physical and mental functions. Soy products also contain a variety of nutrients and bioactive substances, depending on their type, such as fermented/nonfermented and microorganisms involved in the fermentation. Therefore, it is not easy to speculate the mechanism of the effect of the intake of soy products in general on BADL/IADL. However, it is possible to speculate a mechanism related to the results of this study based on several epidemiological studies suggesting the disease-preventive effects of soy isoflavones,^12,13^ vitamin K_2_ in natto (a soybean food unique to Japan fermented with the bacillus natto),^14,15^ and melanoidins in miso and soy sauce (both ancient Japanese foods fermented with yeast).^16,17^

### Isoflavones

Epidemiological studies suggest that isoflavone consumption is effective in preventing cerebral and myocardial infarctions and osteoporosis, especially in postmenopausal women. A prospective cohort study conducted between 1990 and 2002 in Japanese women (aged 40–59 years) found that in postmenopausal women, the multivariate hazard ratios (95% CI) for the highest quintile per lowest quintile of isoflavone consumption were 0.35 (0.21–0.59) for stroke and 0.37 (0.14–0.98) for myocardial infarction, meaning that the risk of cerebral and myocardial infarction was reduced in women who consumed more isoflavones.^12^

A meta-analysis of randomized controlled trials found that soy isoflavones significantly increased bone mineral density by 54% and decreased urinary deoxypyridinoline, a bone resorption marker, by 23%. Particularly large effects were observed in postmenopausal women and at isoflavone doses of 75 mg/day or more.^13^

### Vitamin K_2_

Natto intake on a daily basis may help prevent bone fractures, osteoporosis, and arteriosclerosis. Natto is rich in vitamin K_2_ (menaquinone-7 [MK-7]), which contributes to the carboxylation of osteocalcin, a bone protein involved in bone formation, and to the prevention of arterial calcification.^14^ A study targeting Japanese people found that serum MK-7 levels were significantly higher in those who frequently consumed natto.^15^ The incidence of hip fractures in women tended to be lower in prefectures with higher per capita consumption of natto.^15^ These findings suggest that high MK-7 levels from natto consumption may contribute to lowering fracture risk in Japanese women.

In the Rotterdam Study, a longitudinal study that examined the association between vitamin K_1_ and K_2_ intake and heart disease, arterial calcification was reduced in the group with higher vitamin K_2_ intake.^16^ Mortality by coronary heart disease (CHD) was about half in the middle and upper tertiles of vitamin K_2_ intake than in the lower tertile.^16^ These findings suggest that adequate intake of menaquinone may be effective in preventing CHD. According to the 2010 Comprehensive Survey of Living Conditions, cerebrovascular disease (21.5%), fractures and falls (10.2%), and heart disease (3.9%) account for a large proportion of the factors contributing to the need for nursing care.^17^ The higher intake of vitamin K_2_ may have led to a reduction in BADL/IADL decline in this study.

### Melanoidin

Melanoidin, found in miso and soy sauce, is a brownish substance produced through the “Maillard reaction” that occurs when sugar and amino acids are heated. It is noted for its antioxidant properties, protection of vascular health, and intestinal regulation.^18^ When melanoidin was added to the culture of cells derived from human colon or gastric cancer, a remarkable inhibitory effect against cell growth was observed.^19^ Epidemiological studies on the health effects of melanoidin are lacking, but the effects of melanoidin may partly explain the results of this study, as cancer accounts for 2.3% of the need for nursing care.^17^

### Strengths and limitations

The strength of this study is that it prospectively clarifies how the frequency of soy product consumption affects overall life function parameters such as BADL and IADL, rather than individual diseases. In contrast, there are several limitations in interpreting the results of this study. First, the main independent variable, the frequency of soy product consumption, was examined through interviews. Thus, what was actually examined was each individual's perception on the frequency of soy product consumption. This means that even if two people eat the exact same food with the same frequency, their answers could vary depending on their level of interest in food, or on whether they cook for themselves, and so on.

The second limitation was the generalization among ethnic groups because this study targeted only Japanese people. Particular attention should be paid to ethnicity, as soy product consumption may not necessarily produce the same degree of positive results in other ethnic groups, where a relatively high percentage of people do not have the equol-producing bacteria that maximize the effects of soy isoflavones.^20^ Third, in this study, only those individuals without disabilities, that is, 86% for BADL and 81% for IADL, were included in the longitudinal analysis. Though we do not believe that this jeopardizes the value of the results, the lack of validation in individuals who already have disabilities might be a limitation.

Fourth, there are so many different types of soy products eaten in Japan: natto, tofu, abura-age (deep-fried tofu), miso, soybean flour, soy sauce, soy milk, okara (a byproduct of tofu), yuba (bean curd), roasted beans, and koya-dofu (freeze-dried tofu). It is unknown which of these the participants identified as soy products. The minimum amount to be recognized as consumed is also unknown. Also, although each soy product has different nutritional components, data are only available here on soy products in general. To see the effects of consumption of bioactive compounds specific to individual soy products, it is necessary to investigate the consumption of individual soy products.

## Conclusion

For Japanese women living in the community aged 75 years or older without BADL or IADL disabilities, more frequent soy product intake at baseline was associated with less BADL or IADL disabilities, respectively, after 4 years. This study provides a rationale for planning intervention studies on soy product consumption in older Japanese women.

## Abbreviations Used

ADL: activities of daily living
BADL: basic activities of daily living
BMI: body mass index
CHD: coronary heart disease
CI: confidence interval
IADL: instrumental activities of daily living
MK-7: menaquinone-7
OR: odds ratio
P-DVS: partial dietary variety score
SD: standard deviation
